# Simultaneous Occurrence of Choriocarcinoma in an Infant and Mother

**DOI:** 10.3390/ijerph18041934

**Published:** 2021-02-17

**Authors:** Małgorzata Rzanny-Owczarzak, Joanna Sawicka-Metkowska, Katarzyna Jończyk-Potoczna, Ewelina Gowin, Patrycja Sosnowska-Sienkiewicz, Przemysław Mańkowski, Danuta Januszkiewicz-Lewandowska

**Affiliations:** 1 Department of Pediatric Surgery, Traumatology and Urology, Poznan University of Medical Sciences, 60-572 Poznań, Poland; sosnowska@ump.edu.pl (P.S.-S.); mankowskip@wp.pl (P.M.); 2 Department of Pediatrics, St. Joseph’s Hospital, 61-825 Poznań, Poland; 3 Department of Pediatric Radiology, Poznan University of Medical Sciences, 60-572 Poznań, Poland; potocznak@op.pl; 4 Department of Health Promotion, Poznan University of Medical Sciences, 60-781 Poznań, Poland; ewego@ump.edu.pl; 5 Department of Pediatric Oncology, Hematology and Transplantology, University of Medical Sciences, 60-572 Poznań, Poland; danuta.januszkiewicz@ump.edu.pl; 6 Department of Medical Diagnostics, University of Medical Sciences, 60-569 Poznań, Poland

**Keywords:** choriocarcinoma, gestational trophoblastic disease, infant, infantile choriocarcinoma, newborn

## Abstract

Infantile choriocarcinoma is an extremely rare disease. We present a case study of a 1-month-old male with choriocarcinoma diagnosed simultaneously with his mother. On admission to hospital, the disease was very advanced and massive progression and multi-organ failure caused the death of the patient despite the implemented treatment. It was too late to save the child’s life, but early enough to save his mother. The authors believe that the serum levels of hCG should be determined in every newborn with anemia and liver tumor, especially when the mother has a positive history of miscarriage.

## 1. Introduction

Primary infantile or neonatal choriocarcinoma is an extremely rare disease and the most cases involve metastatic disease to the fetus from an intraplacental chorioncarcinoma [1]. When not diagnosed and untreated, the disease has always been fatal [2]. In disseminated infantile choriocarcinoma, the use of intensive chemotherapy resulted in the possibility of saving neonatal life in 20% of cases. In the remaining children, an infantile choriocarcinoma is still associated with poor prognosis and high mortality.

Retrospective analysis of infantile choriocarcinoma showed that maternal choriocarcinoma was diagnosed in approximately 60% of these infants. Most of these mothers were asymptomatic during pregnancy, even though primary intraplacental choriocarcinoma was developed in uncomplicated third-trimester placentas. Only the development of disease in the mother or infant retrospectively confirmed intraplacental choriocarcinoma [3].

Chorioncarcinoma (CC) next to the pre-malignant conditions like complete or partial hydatiform moles and the malignant ones like placental trophoblastic tumor (gestational trophoblastic tumor) belongs to gestational trophoblastic disease (GTD). The exact frequency of CC is less ambiguous than GTD, but some data shows the incidence to be around 1:50,000 deliveries [4]. In the United States for example, CC is estimated to affect approximately 2 to 7 of every 100,000 pregnancies [5].

All forms of GTD come from the elements of the normal human placenta, but the exact pathogenesis of choriocarcinoma has not been fully explained. Choriocarcinoma develops from abnormal trophoblastic population cells with hyperplasia and anaplasia. CC is characterized not only by a proliferation of cytotrophoblasts and syncytiotrophoblasts but also by an extensive angioinvasion of myometrium [6,7]. Histologically, choriocarcinoma shows an absence of chorionic villi [8].

There are two forms of choriocarcinoma: gestational and non-gestational. Most of them are gestational in origin, while non-gestational ones are exceedingly rare. [9]. The gestational one mostly arises following a hydatidiform mole, but can be also observed during and after normal pregnancy, or most commonly after abortion. The non-gestational choriocarcinoma arises from pluripotent germ cells and is observed very rarely either in a population of young men or of young women. Although histologically and morphologically is similar to pregnancy-related CC, their genetics and immunogenicity are different. Prognosis is worse mainly due to lower sensitivity to chemotherapy [10].

The exact diagnosis, staging, risk assessment and treatment depend on the type of GTD and the level of hCG are given in the clinical practice guidelines [4]. Nevertheless, every woman who experienced a molar pregnancy should be counseled as to the risk for development of choriocarcinoma. These patients should be monitored closely for their hCG levels.

We present a case study of a 1-month-old male with choriocarcinoma diagnosed simultaneously with his mother. On admission to hospital, the disease was very advanced and massive progression and multi-organ failure caused the death of the patient despite the implemented treatment. It was too late to save the child’s life, but early enough to save his mother. The authors believe that in every newborn with anemia and liver tumor, the serum levels of hCG should be determined, especially when the mother has a positive history of miscarriage.

## 2. Case Study

A 1-month-old male Caucasian was transferred from the local pediatric ward to the Department of Pediatric Oncology, Hematology and Transplantology because of a suspected liver tumor. In children at this age, hepatoblastoma is the third most common (after neuroblastoma and nephroblastoma) malignant tumor of the abdomen. This was therefore the first suspected diagnosis. Vomiting with traces of blood was observed twice in the two preceding days before admission. In the pediatric ward, anemia was diagnosed and an abdominal ultrasound revealed a liver tumor with rich vascularization. The patient was a full-term neonate, born from a 3rd pregnancy, the 2nd birth, Apgar 10, with a birth weight 3095 g and was a caesarean section due to a lack of progress in delivery. The first pregnancy was complicated by gestational diabetes in the mother, and the second one ended in a miscarriage in the 3rd month of pregnancy (that was 4 months before the third pregnancy).

**Figure 1:**
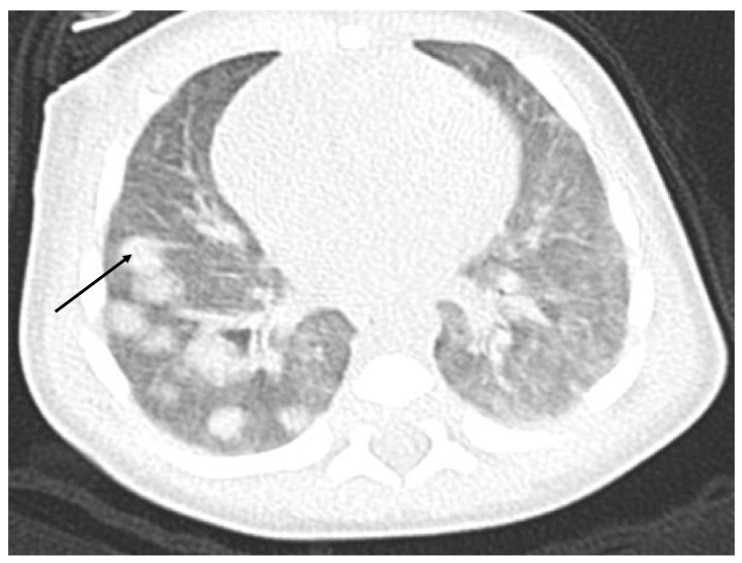
Chest CT with numerous metastatic lesions in both lungs.

The ultrasound examination performed on admission in the oncological ward showed a massive hypodensive lesion in the right lobe of the liver, which was 5 cm in diameter. The lung X-ray indicated three circular metastatic lesions. Chest and abdominal computed tomography (CT) showed numerous (about 50) metastatic lesions in both lungs, and a liver tumor 5.2 cm × 3.7 cm × 4.8 cm in the right lobe. Lung window axial CT image shows multiple pulmonary rounded nodules of variable size, scattered throughout both lungs (Figure 1).

**Figure 2:**
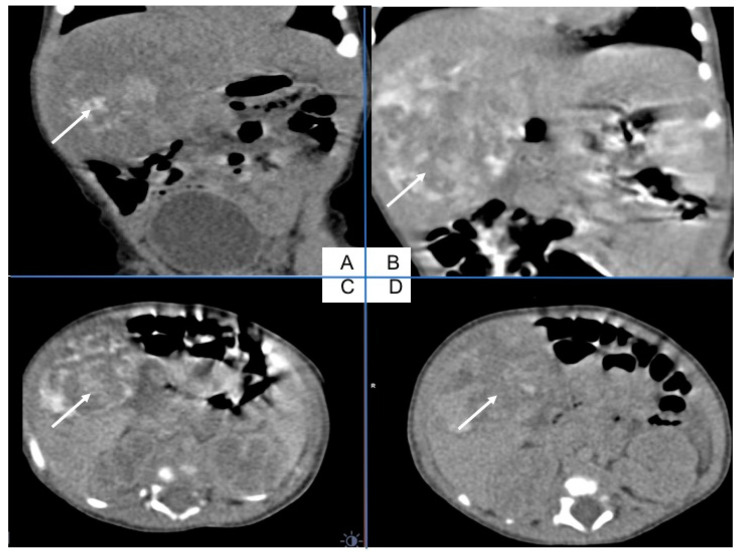
Abdominal CT with liver tumor in the right lobe. An abdominal nonenhanced (**A**) and enhanced arterial phase (**B**) coronal view, arterial phase (**C**) and portal phase (**D**) axial view indicate on the hepatic mass with bleeding (**A**), and central necrosis. After gadolinium contrast injection, there is poor peripheral enhancement in the arterioportal phase (**C**) and in the late portal venous phase CT image shows poor hypodense lesions (**D**).

An abdominal nonenhanced (A) and enhanced arterial phase (B) coronal view, arterial phase (C) and portal phase (D) axial view indicate on the hepatic mass with bleeding (A), and central necrosis (Figure 2). After gadolinium contrast injection, there is poor peripheral enhancement in the arterioportal phase (C) and in the late portal venous phase, the CT image shows poor hypodense lesions (D) (Figure 2). 

An abnormal blood tests were anemia (Hb 6.1 g/dL), thrombocytopenia (77.000/uL), high BNP (1259 pg/mL), elevated total bilirubin (1.71 mg/dL), elevated LDH (651 IU/L), low fibrinogen (88 ng/dL), AFP (1110 ng/mL, but within the age limit) and an extremely high hCG level (2,813,000 IU/mL).

The results of the CT and hCG tests authorized the urgent start of chemotherapy. A central catheter had to be inserted before treatment. Unfortunately, after surgery, due to significant respiratory efforts against tachypnoe and tachycardia, the infant was transferred to the ICU and chemotherapy with ethoposide (100 mg/m^2^ with a dose reduction acc. to the body weight) was started. Despite two days of chemotherapy and intensive treatment, the clinical condition deteriorated rapidly, massive malignant progression in the lungs (X-rays), in abdomen with intestinal and lymph nodes infiltration (USG), as well as in the CNS (USG) was observed. In fourth day of hospitalization, the patient died due to multiple organ failure. The post-mortem histopathological examination confirmed the diagnosis of disseminated choriocarcinoma.

The child was in the ward with his father. The mother stayed at home because of vaginal bleeding. After receiving the results of the child’s hCG, the mother was urgently requested to the gynecological ward, where after curettage, a hydatidiform mole/choriocarcinoma was diagnosed (mother’s hCG was 37,600 IU/mL). The mother was treated with chemotherapy for 6 months (dactinomycin, etoposide, methotrexate acc. to FIGO -International Federation of Gynecology and Obstetrics protocol). Currently, one year after the occurrence of GTD, despite being advised not to conceive until her follow-up is complete, she is pregnant again.

## 3. Discussion

The occurrence of choriocarcinoma in infancy (infantile choriocarcinoma) is an immensely rare. The age of the presented patient was typical for infantile choriocarcinoma manifestation. First symptoms of infantile choriocarcinoma usually occurs at a median age of 1 month [11].

Typical early symptoms are anemia, developmental delay, hepatomegaly, hemoptysis or respiratory failure, while there may be also signs of precocious puberty. Our patient was admitted to the hospital because he presented vomit stained with blood. Physical examination showed an enlarged liver and in laboratory tests, anemia was found. Abdominal ultrasound examination revealed a tumor with rich vascularization. Because hepatoblastoma is the most common primary liver tumor in children and is usually diagnosed during the first years of life, suspicion of this tumor has come to the fore. Further imaging and deterioration of the child’s condition forced increased investigations. AFP level was in normal range according to age, which could rule out diagnosis of hepatoblastoma while an extremely high hCG level indicated CC. Such an elevated level of human chorionic gonadotropin is strongly typical for metastatic CC. In all 30 cases reported in Blohm and Göbel’s study, hCG was always elevated [11]. As Getrajdman et al. claim, isolated elevation of hCG in the presence of liver mass has been reported only in choriocarcinoma [12]. Choriocarcinoma can spread directly through the vessels and the middle layer of the uterine wall and cover distant areas such as the vagina, adnexa, lungs, spleen, intestines, kidneys and liver [13]. It also reached a distant places such as the fetus. The localization of CC in the neonatal liver can be explained by prenatal blood supply rules. Kiserud T et al. in their studies show that the fetal liver receives 70% to 80% of the umbilical cord in the second half of a human pregnancy, leaving only 20% to 30% to be shunted through the ductus venosus [14]. This may be the reason why placenta CC primarily gives bloodstream metastases to the fetus’ liver. Further, Blohm in their study describe that in 73% of the reported neonatal or infantile choriocarcinoma cases, the liver was involved [15]. Traboulsi W et al. proved in a mouse model that the placental environment and vascularization contribute to CC metastasis to various organs, including the liver [16].

However, in some cases the liver may be the primary site of the choriocarcinoma as well [17,18]. Yet, the location in the right, not in the left lobe of the child’s liver, the higher incidence of distant metastases [19] and, above all, choriocarcinoma of the mother’s placenta, argues for the origin of choriocarcinoma from the mother to the fetal liver and not the primary liver tumor. At the first breath at birth, the resistance in the lung vessels decreases and blood begins to circulate in the pulmonary vessels. Lung tumors are hematogenous metastases after opening of the pulmonary circulation after birth. In 65% of children, the presence of metastases in the lungs during CC was found [15]. Post-mortem diagnosis is typical for infantile choriocarcinoma [11]. In our case, although CC was recognized, the detailed diagnosis was also established in the post-mortem examination.

A history of miscarriage increases the chances of having molar pregnancy, and hence choriocarcinoma as well. Genetic disorders of gamets might be the basis for both miscarriages and mole pregnancy. For example, diandric triploids are the most frequent chromosome abnormality in infertile males with oligo-, crypto- and azoospermia [20]. Moreover, the woman was also not tested for the NLRP7 mutation, which is a major gene responsible for recurrent hydatiform moles. Due to the difficulty in diagnosing a molar pregnancy before evacuation, it is recommended that, in failed pregnancies, products of conception must be examined histologically [21]. In this case, unfortunately, this has not happened. Moreover, the authors of the Clinical Guidelines in GTD noted that intraplacental CC is rarely discovered, probably because placentas are not routinely sent for pathological examination, so their true frequency is difficult to specify. When it is discovered is probably the source of metastatic disease after term pregnancies [7].

The most frequently observed symptom of the maternal disease is intermittent vaginal bleeding after delivery [22], which also occurred in this case. Symptoms from metastatic disease, such as dyspnoea or neurological disorders, can occur very rarely. Usually the placenta is considered to be the cause of CC, but in our case, maternal excrecta has not been histologically examined after delivery. Most maternal cases of CC occur within a few months or even years after delivery and are usually metastasized. In our case, fortunately, the rapid diagnosis of the mother has allowed CC to be diagnosed without metastatic features and to be completely cured.

Women undergoing chemotherapy are advised to avoid pregnancy for 1 year after completing the chemotherapy. During that year, patients are at high risk of the disease recurrence. In addition, a physiological increase of hCG levels during pregnancy may make it difficult to detect and diagnose CC relapse as early as possible. However, women who become pregnant and desperately want to have a baby can be sure of a likely successful outcome [23].

## 4. Conclusions

Although the incidence of CC in infants is extremely rare, about a quarter of them show symptoms after birth and the diagnosis can be easily confirmed with hCG. The authors recommend that in every newborn with anemia and liver tumor, the routine hCG testing should be offered both for an infant and for a mother, especially when the mother has a positive history of miscarriage. In addition, particular emphasis should be placed on the histopathological examination of excrecta after miscarriages and deliveries, especially since GTD and intraplacental CC can be easily underdiagnosed because most women are asymptomatic with a macroscopically normal placenta.
